# A diffusion-based quantification technique for assessment of sacroiliitis in adolescents with enthesitis-related arthritis

**DOI:** 10.1259/bjr.20150775

**Published:** 2016-01-15

**Authors:** Kanimozhi Vendhan, Timothy J P Bray, David Atkinson, Shonit Punwani, Corinne Fisher, Debajit Sen, Yiannakis Ioannou, Margaret A Hall-Craggs

**Affiliations:** ^1^ Academic Radiology, University College London Centre for Medical Imaging, London, UK; ^2^ Arthritis Research UK Centre for Adolescent Rheumatology, University College London, London, UK

## Abstract

**Objective:**

To investigate the use of a quantitative diffusion-weighted imaging (DWI) tool for measuring inflammation of the sacroiliac joints (SIJs) in enthesitis-related arthritis (ERA).

**Methods:**

A retrospective study was performed with institutional review board approval. Subjects were adolescents who had undergone MRI of the SIJs since January 2010. 10 patients with a clinical diagnosis of ERA and 10 controls with a clinical diagnosis of mechanical back pain were assessed. Axial *T*_1_ weighted, short tau inversion recovery (STIR) and DWI (*b*-values 0, 50, 100, 300 and 600 mm^2^ s^−1^) images were acquired. Apparent diffusion coefficient (ADC) maps were generated using a monoexponential fit. On each of four slices, two to three linear regions-of-interest were placed on each joint. Normalized ADC (nADC) values were defined as joint ADC divided by a reference ADC derived from normal sacral bone. STIR images were scored using a modification of an established technique. The correlation between nADC values and STIR scores was evaluated using Spearman's rank correlation.

**Results:**

Mean nADC values were significantly higher in cases than in controls (*p = *0.0015). There was a strong correlation between STIR scores and nADC values (*R* = 0.85).

**Conclusion:**

ADC values are significantly increased in inflamed SIJs compared with controls. There is a good correlation between this diffusion-based method and STIR scores of inflammation.

**Advances in knowledge:**

We have described and provisionally validated a method for quantifying the severity of inflammation in the SIJs in ERA using ADC measurements. This method is quick, is reproducible and could potentially be automated.

## INTRODUCTION

Juvenile idiopathic arthritis is the most common form of arthritis in children and adolescents and is a significant cause of morbidity.^1^ Enthesitis-related arthritis (ERA) is a disease subtype that is more common in adolescents and harbours particularly poor outcomes; compared with other subtypes, patients with ERA experience poorer long-term physical health and more disability.^2^ ERA is difficult to assess clinically owing to the fluctuating nature of the disease and because adolescents are poor reporters of pain.^3^ Patients often experience prolonged delays in diagnosis.^4^ Spondyloarthritis with inflammation of the sacroiliac joints (SIJs) is common in ERA and is strongly associated with human leukocyte antigen-B27 positivity; sacroiliitis is present in approximately 80% of patients with ERA with inflammatory low back pain but may go undetected using clinical methods alone.^5^

Biologic therapies such as etanercept are an effective treatment for ERA but come with a risk of immunosuppression and more rarely myelitis and optic neuritis.^6,7^ There is a need for objective markers of joint inflammation to inform therapeutic decision-making and also to facilitate clinical trials of new therapeutic agents.

The Spondyloarthritis Research Consortium of Canada has developed a validated scoring system for the assessment of disease activity in the SIJs in adult patients with ankylosing spondylitis using short tau inversion recovery (STIR) images.^1^ However, this system is subjective and only offers the scorer a binary choice for each joint quadrant—a tiny patch of inflammation within one quadrant may receive the same score as inflammation occupying the whole quadrant. Furthermore, there is no assessment of inflammation in the joint itself or the ligamentous joint. Additionally, the technique does not measure erosions or fatty change and has not been validated in children or adolescents.

Diffusion-weighted imaging (DWI) offers a new approach to assessing inflammation. Inflammation produces an increase in the apparent diffusion coefficient (ADC) of water molecules in affected tissues, probably owing to an increase in the ratio of extracellular to intracellular water.^8–10^ DWI has been used in the assessment of sacroiliitis in ankylosing spondylitis^11–13^ and is promising as a potential biomarker of disease activity in juvenile idiopathic arthritis.

A key objective of the current study was to develop an imaging “biomarker” of inflammation in ERA, which reflects *overall* activity in the SIJs and could be used to guide treatment. ERA is a disease that fluctuates, and one area of inflammation can improve as another deteriorates. Consequently, serial measurements of focal areas of disease do not reflect overall disease activity. Studies in adult sacroiliitis have typically placed regions of interest (ROIs) on regions of inflammation which were identified by the observer^12^ or have used small, circular ROIs on subchondral bone.^11,13^ However, we felt that these tools inadequately reflected inflammation in the whole joint, which clearly also involves the joint space and synovium. We have therefore developed a practical linear ROI tool for measuring joint inflammation. This tool provides a “cross-section” of the joint which includes relatively constant proportions of joint space, synovium and subchondral bone.

In this study, we evaluate the use of this quantitative tool for measuring inflammation of the SIJs in ERA using DWI. It is hypothesized that ADC values will be increased in sacroiliitis compared with controls and that ADC values will correlated with STIR scores.

## METHODS AND MATERIALS

This retrospective study was covered by institutional review board approval from the National Research Ethics Service Committee London, Bentham, England (REC ref: 11/LO/0330). Informed consent was obtained for review of all clinical investigations.

### Subjects

A picture archiving and communication system search was performed to identify all those adolescents who had MRI of the SIJs since January 2010. All patients had attended a specialist adolescent rheumatology clinic at Arthritis UK Centre for Adolescent Rheumatology (UCL). “Cases” were patients who fulfilled the International League of Associations for Rheumatology criteria for ERA.^14^ “Controls” were patients with a final diagnosis of mechanical back pain (*e.g.* owing to disc disease or pars interarticularis fractures), with inflammatory markers [ESR and C-reactive protein (CRP)] within the normal range. The first 10 cases and 10 controls those fulfilled these criteria were selected. The ages of the two groups were compared using a two-sample *t*-test.

### MRI technique

MRI of the SIJs was performed using a 1.5-T system (MAGNETOM® Avanto; Siemens Healthcare, Germany) with an integrated phased array spine coil, together with anterior phased array body coil. Scan parameters were as follows:

*T*_1_ turbo spin echo (TSE) coronal—repetition time/echo time (TR/TE), 610/11 ms; slices, 18; slice thickness, 3 mm; field of view (FOV), 200 mm
*T*_1_ TSE axial—TR/TE, 475/11 ms; slices, 20; slice thickness, 5 mm; FOV, 200 mm
STIR axial—TR/TE, 6070/83 ms; slices, 18; slice thickness, 5 mm; FOV, 200 mm
*T*_1_ turbo inversion recovery magnitude coronal—TR/TE, 4340/83 ms; slices, 14; slice thickness, 4 mm; FOV, 200 mm
STIR axial—TR/TE, 6070/83 ms; slices, 18; slice thickness, 5 mm; FOV, 200 mm
Post-contrast *T*_1_ TSE fat sat axial—TR/TE, 619/11 ms; slices, 20; slice thickness, 5 mm; FOV, 200 mm
Post-contrast *T*_1_ TSE fat sat coronal—TR/TE, 795/11 ms; slices, 18; slice thickness, 3 mm; FOV, 200 mm
Single-shot diffusion-weighted images with echo planar readout—TR/TE, 3500/87 ms; FOV, 316; slice thickness, 8 mm; averages, 4; EPI factor, 120; *b*-values: 0, 50, 100, 300 and 600 s mm^−2^ with fat saturation.

ADC maps were generated on standard vendor software using a mono-exponential fit.

### Image analysis

**Figure 1.**
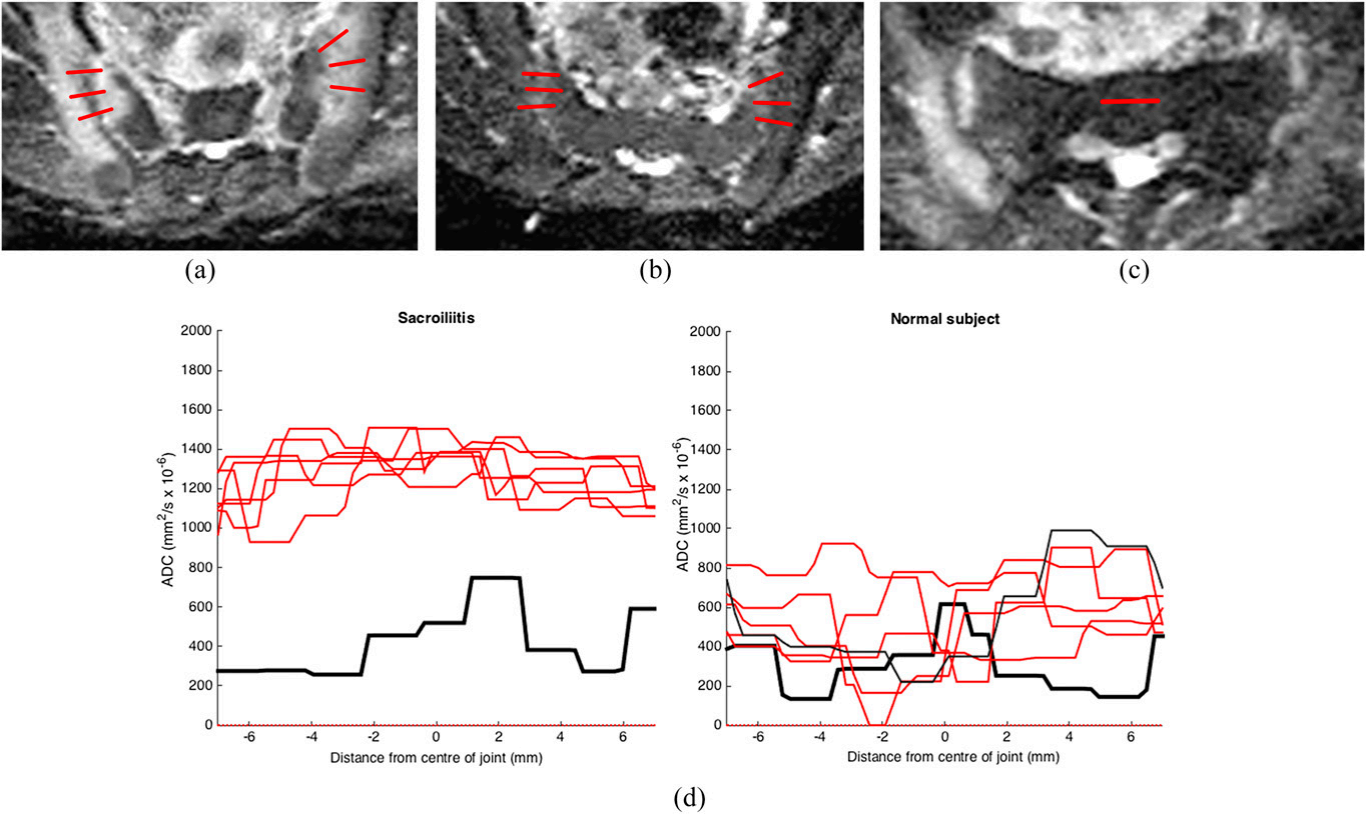
Placement of linear regions of interest (ROIs) (red) shown on the apparent diffusion coefficient (ADC) map of a patient with enthesitis-related arthritis and sacroiliitis (a) and a control with mechanical back pain (b). Only one slice is shown for each patient (four slices were scored in total). (c) The reference ROI that was placed on interforaminal sacral bone and used to generate a reference profile. (d) ADC profiles from the two subjects (in red) are shown compared with the reference profile from interforaminal sacral bone (in black). The six ADC profiles correspond to the ADC profiles shown in (a) and (b).

Axial *T*_1_ weighted, STIR and DWI images of the SIJs were anonymized using DicomCleaner™ (PixelMed Publishing™, Bangor, PA). The central four axial images on the ADC maps (*i.e.* those that best represented the synovial portion of the SIJ) were analysed using in-house MATLAB^®^ (MathWorks^®^, Natick, MA) code. Two to three linear ROIs measuring 14–16 mm were drawn across the synovial portion of the SIJ, with each ROI centred on the joint space (Figure 1). The decision to use two or three ROIs was made based on the anteroposterior dimensions of the joint; where the joint was too short to accommodate three ROIs, only two were used. Both observers placed the same number of ROIs on each slice (the second observer was given slice numbers and the number of ROIs in advance but was blinded to exact ROI placement). Similarly, ROIs were omitted from regions where there was an artefact overlapping the joint. The number of ROIs for each patient was recorded. A further “reference” ROI was placed on normal sacral bone to provide internal standardization. The software was used to generate a profile of ADC values along each of the linear ROIs (Figure 1). The normalized ADC (nADC) value of each patient was defined as the ratio between the mean ADC of all joint line profiles and the mean ADC of the reference profile. To assess inter- and intraobserver variability, two observers (KV and TJPB) each performed two sets of readings 2 months apart in all subjects.

The contrast-enhanced images were not analysed in the study itself, although the clinical reports from the images may have played a role in the diagnosis of ERA, which was made according to International League of Associations for Rheumatology criteria.^14^

### Validation of the diffusion-weighted imaging tool

The ADC quantification technique was validated by comparison with a STIR-based quantification method. The Spondyloarthritis Research Consortium of Canada technique^1^ was modified to use axial rather than coronal images, to facilitate direct comparison with axial ADC maps. The slice thickness of ADC maps was 8 mm, in order to generate images with sufficient signal. Four ADC slices (8 mm) and six STIR slices (5 mm) were analysed, so that an equivalent volume of bone was evaluated (32/30 mm). The slices were matched anatomically.

The STIR scoring was performed by two readers, with 5 and over 20 years' of experience of musculoskeletal MRI, who were blinded to the clinical data and the other reader's scores. On each axial STIR slice, the SIJ was divided into four quadrants: anterior and posterior iliac, and anterior and posterior sacral. Increased STIR signal was given a score of 1 per quadrant and normal signal was scored 0. For each slice, an additional score of 1 per joint was given if a lesion exhibited intense signal (equal or greater than the signal of nearby blood vessels) and 1 per joint if a lesion had ≥1-cm depth from the articular surface. The maximum score in one slice is 12 and in six slices is 72. The mean average score of the two observers was used for the analysis.

### Statistical analysis

An unequal variances *t*-test (Welch test) was used to compare nADC values between cases and controls. The inter- and intraobserver variation in nADC scores was assessed using Bland–Altman plots (95% limits of agreement) and intraclass correlation coefficient (absolute agreement between measurements).^15^ Spearman's rank correlation was used to examine the association between the STIR scores and normalized ADC value. The mean STIR score from the two observers was used in the analysis.

### Sacroiliac joint maturation

SIJ maturation was assessed in all subjects. Subjects with open sacral segmental apophyses were deemed to be immature.^16^

## RESULTS

### Demographics

The mean age of the cases was 16 years (range 11.9–22.4 years) and of the controls was 14.6 years (range 9.6–14.6 years). There was no significant difference between the ages of the two groups (*p* = 0.25). All 10 cases were male (reflecting the male predominance of the disease), whereas the controls consisted of 7 males and 3 females.

### Regions of interest

For cases, the mean number of ROIs was 20.4 [range 16–24, standard deviation (SD) 2.63]. For controls, the mean number of ROIs was 18 (range 16–22, SD 1.63).

### Normalized apparent diffusion coefficientin cases and controls

**Figure 2.**
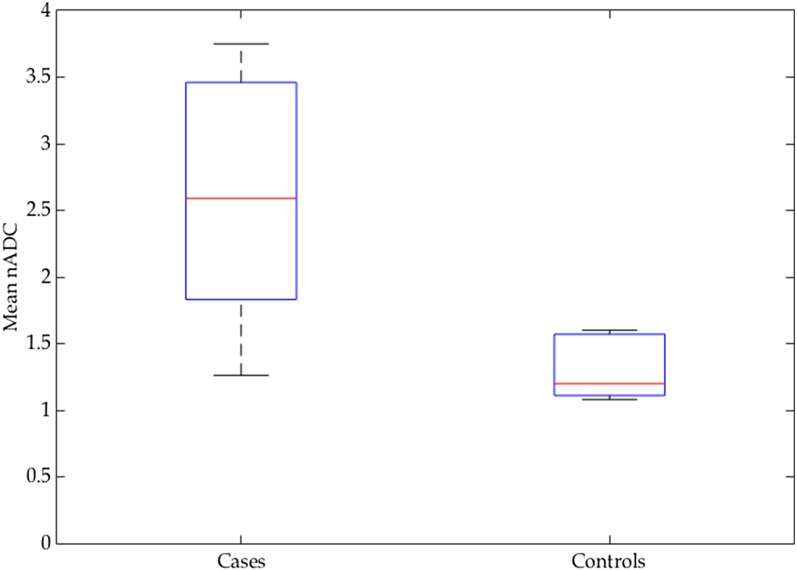
Box plot comparing normalized apparent diffusion coefficient (nADC) values in cases and controls. nADC values were significantly higher in cases (mean 2.57) than in controls (mean 1.28) (*p* = 0.0015).

**Figure 3.**
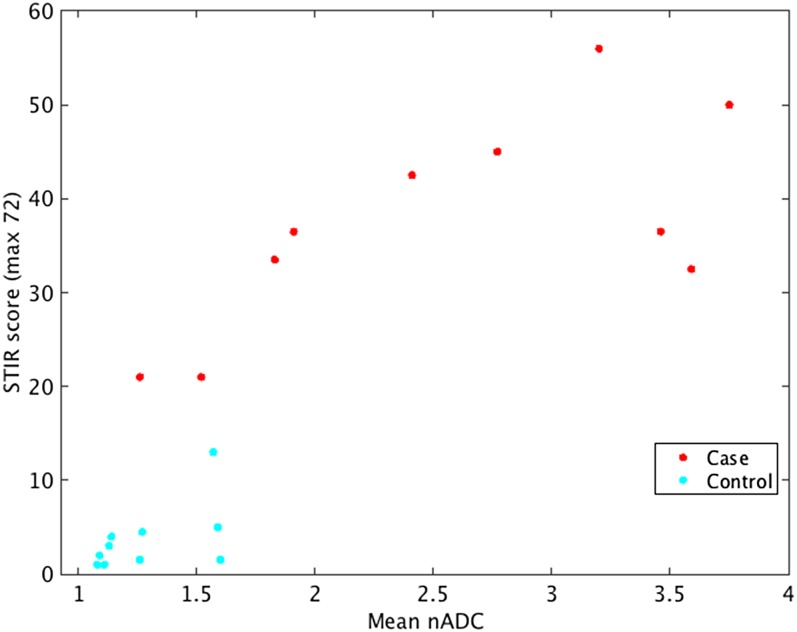
Scatterplot comparing mean normalized apparent diffusion coefficient (nADC) and short tau inversion recovery (STIR) scores in cases (*n* = 10) and in control subjects (*n* = 10). STIR and nADC values were strongly correlated (*R* = 0.85, *p* < 0.001).

Mean nADC values were significantly higher in cases (mean 2.57, SD 0.91) than in controls (mean 1.28, SD 0.22) (*p* = 0.0015) (Figure 2). There was a strong correlation between STIR scores and nADC values (*R* = 0.85, *p* < 0.001) (Figure 3).

### Interobserver agreement

Interobserver agreement was assessed across all patients (both ERA cases and controls) for the first set of measurements by the two readers. The mean difference between the two sets of measurements was 0.15. The Bland–Altman 95% limits of agreement was ±1.75 across a range of values from 1.08 to 3.75. The intraclass correlation coefficient was 0.64.

### Intraobserver agreement

For observer 1 (KV), the mean difference between measurements was −0.14, with Bland–Altman 95% limits of agreement at ±1.6 across a range of values from 1.02 to 4.30. The intraclass correlation coefficient was 0.73. The within-subject coefficient of variation was 42%.

For observer 2 (TJPB), the mean difference between measurements was 0.1, with Bland–Altman 95% limits of agreement at ±0.9 across a range of values from 1.09 to 4.63. The intraclass correlation coefficient was 0.87. The within-subject coefficient of variation (SD/mean) was 24%.

### Outliers

There was a small overlap in mean nADC values for cases and controls. Two ERA cases were judged to be outliers at the lower end of the nADC scale; these were case 56 (nADC* = *1.26) and case 65 (nADC* = *1.52). At the upper end of the control distribution, the two highest nADC values belonged to control 28 (nADC* = *1.57) and control 36 (nADC* = *1.60). Review of the MR images of these patients showed that the two cases had only mild sacroiliitis, which was reflected in their STIR scores (STIR score* = *21 in both cases). Controls 28 and 36 were adolescents with particularly immature SIJs as evidenced by open sacral segmental apophyses.^16^

Assessing intraobserver variability, the largest individual difference between a single observer's scores was for case 63 (KV; nADC difference* = *2.55). In this case, there was a large change in the mean reference ADC value between the two repeat scores for this observer (120* *mm^2^ s^−1^ compared with 278* *mm^2^ s^−1^). Similarly for case 66 (KV; nADC difference* = *2.05), the mean reference ADC values were 333 and 194* *mm^2^ s^−1^.

## DISCUSSION

In this study, we have described and provisionally validated a method for quantifying the severity of inflammation in the SIJs in ERA using ADC measurements. We have shown that ADC values are significantly increased in inflamed SIJs compared with controls (*p *<* *0.01). There is a good correlation between this diffusion-based method and STIR scores of inflammation based on a modification of an established technique (*R *=* *0.85).^1^

The intra- and interobserver reproducibility for mean nADC scores were good.^17,18^ However, the interobserver variability was greater than intraobserver variability, possibly owing to subtle differences in the way the linear ROIs were placed by different observers. In cases where a single observer's second score was very different to the first, variation in the reference ADC accounted for much of the variability.

nADC values of cases with mild inflammation were similar to control patients with immature SIJs (*i.e.* open segmental apophyses^16^). This may be due to the higher proportion of unossified cartilage in immature subjects compared with mature subjects.

The relationship between changes in tissue water content and ADC is well established.^19^ The increase in ADC values with inflammation is thought to be due to an increase in extracellular water, which causes an increase in the average diffusivity of water molecules in a particular tissue.^8–10^ Our results are consistent with adult studies demonstrating that ADC is elevated in sacroiliitis^11–13^ and studies in children showing that osseous oedema in chronic non-bacterial osteomyelitis causes increased ADC.^8^

If ADC values can be shown to change with treatment, DWI could be used to monitor response to therapy and guide management decisions. This technique could also help to determine the efficacy of different drugs used in clinical trials—for example, disease-modifying antirheumatic drugs and antitumour necrosis factor inhibitor drugs. Although the results indicate a significant association between STIR scores and normalized ADC values at baseline, it is not currently possible to comment on whether the ADC and STIR scores stay coherent with treatment.

There are a number of technical factors that limit the reproducibility of diffusion-based measurements. This is partly due to intrinsic variability in ADC values between scans and across imaging platforms. The quality of fat suppression influences measured ADC values, since fat ADC values are much lower than water ADC values.^20^ The chosen range of *b*-values is also important; at low *b*-values, the diffusion effect may be overestimated because perfusion effects can also cause attenuation of the signal, whereas at high *b*-values (greater than 600 s mm^−2^) diffusion effects may be underestimated because signal intensities are comparable to background noise.^19,21^ ADC values can also vary spatially due to gradient non-linearities; the use of gradient field maps can reduce error and improve reproducibility substantially, but this is time consuming and not widely available at present.^22^

ADC normalization is designed to reduce the effect of these factors by providing an internal reference standard, and there is a growing body of evidence which suggests that ADC normalization can help to improve reproducibility^23–25^ and diagnostic accuracy.^26^ If DWI is to be used as a quantitative tool for measuring inflammation in multiple centres (and in clinical practice), we suggest that nADC is likely to be a more practical biomarker than uncorrected ADC measurements.

An additional consideration is that ADC measurements depend on the composition of red and yellow marrow, since ADC values are lower in fat than in water, and therefore lower in yellow marrow than in red marrow.^27^ We would therefore expect a gradual decrease in joint ADC values as normal subjects mature. Nonetheless, a major aim of this technique is to perform comparisons before and after treatment, which would typically be over a relatively short time frame relative to changes in bone marrow composition. We expect that the *course* of nADC measurements over time is likely to be a particularly useful marker, since it will trend downwards in normal individuals but increase owing to inflammation in patients with sacroiliitis. To improve the interpretation of nADC measurements in cases of subtle sacroiliitis, it may be necessary to define the normal range for nADC in different age groups/patients with differing levels of skeletal maturity.

An additional limitation of this study is that the ligamentous portion of the joint has not been scored. As enthesopathy is a key feature of ERA, the ligamentous portion of the joint may also be inflamed. The degree to which involvement of the ligamentous joint is important for disease prognosis remains uncertain in ERA.

There is scope for improvement in terms of reproducibility for this technique. A problem with the linear-ROI approach is that subtle differences in placement between observers may affect measured values (for example, if the linear ROI takes a more oblique course across the joint). In future work, we hope to develop software that allows the observer to define the joint space and then automatically draws a number of perpendicular ROIs across the joint. This would help to reduce sampling error and improve reproducibility. In the long term, these measurements could potentially be automated, which would speed up the measurement process significantly.

Further validation of this method of measuring inflammation is necessary in larger numbers of patients with correlation to clinical scores [such as physician global assessment of disease activity, parent/patient assessment of well-being, active joint count and serological markers of inflammation (ESR and CRP)]. Measures of disease activity in adult ankylosing spondylitis (such as bath ankylosing spondylitis disease activity index or ankylosing spondylitis disease activity score) remain yet to be validated in children and adolescents with axial ERA and also measurement of response of disease to treatment.

nADC values are elevated in inflamed SIJs and correlate well with STIR scores of inflammation. nADC could be used as a practical objective biomarker of inflammation and could be used to guide anti-tumour necrosis factor therapy in the clinic. This technique could also be used to assess the efficacy of novel therapies in ERA.

## FUNDING

This work was undertaken at University College London Hospitals/University College London, which receives funding from the Department of Health's the National Institute for Health Research (NIHR) Biomedical Research Centre (BRC) funding scheme. The views expressed in this publication are those of the authors and not necessarily those of the UK Department of Health. MAH-C, SP, TJPB and YI are supported by the NIHR University College London Hospitals BRC, and YI is also supported by Arthritis Research UK Grant 20164.
